# A Divergent TaqMan RT-qPCR Strategy for Isoform-Resolved Detection of HIV-1 Circular RNAs

**DOI:** 10.3390/mps9030077

**Published:** 2026-05-13

**Authors:** Christopher Mauer, Sean Paz, Massimo Caputi

**Affiliations:** Department of Biomedical Science, Charles E. Schmidt College of Medicine, Florida Atlantic University, Biomed-BC71, Boca Raton, FL 33431, USA; cmauer2014@fau.edu (C.M.); spaz@health.fau.edu (S.P.)

**Keywords:** circRNA, HIV-1, TaqMan, qPCR

## Abstract

The HIV-1 genome is initially transcribed as a single primary RNA that undergoes extensive splicing to produce over 40 linear and 15 circular RNA (circRNA) isoforms sharing common sequences. Conventional methods for circRNA detection, such as Northern blotting and hybridization-based assays, are inadequate for distinguishing specific circRNA isoforms when multiple circular and linear species originate from the same transcript. We previously identified 15 HIV-1 circRNAs generated by backsplicing and demonstrated that some enhance viral replication by sequestering cellular miRNAs. PCR-based approaches using divergent primers (RT-qPCR) offer greater specificity for detecting individual circular RNAs under these conditions. Building on this, we have developed a TaqMan qPCR assay capable of specifically detecting 14 HIV circRNA isoforms using backsplicing junction-directed divergent primers coupled to a hydrolysis probe for signal confirmation. Compared with matched SYBR Green assays, the TaqMan platform showed lower background in non-infected controls and reduced variance across donor-derived samples. This method provides a robust platform for selective and qualitative analysis of HIV-1 circRNAs.

## 1. Introduction

Circular RNAs (circRNAs) are covalently closed RNA molecules generated by a non-canonical splicing reaction in which a downstream 5′ splice donor is joined to an upstream 3′ splice acceptor [1]. This process, termed backsplicing, produces a back-splice junction (BSJ) that is absent from the corresponding linear transcript and therefore serves as the defining molecular feature for circRNA identification. Because circRNAs lack free 5′ and 3′ ends, they are resistant to exonucleases that degrade linear RNAs; a property that contributes to their stability and which has made them attractive candidates for biomarkers and therapeutic targets [2].

The defining structural properties of circRNAs also create distinct analytical challenges. In contrast to linear RNAs, circRNAs often share most of their sequence with cognate linear transcripts, differing only by the presence of the BSJ. As a result, methods that rely on transcript size, hybridization to internal exonic sequence, or bulk RNA abundance are often insufficient to discriminate circular from linear RNA species, particularly when multiple isoforms arise from the same transcriptional unit. Accurate circRNA detection therefore requires assay designs that directly interrogate the BSJ and minimize amplification or detection of related linear RNAs. For this reason, circRNA studies frequently combine biochemical enrichment strategies, such as RNase R treatment, with reverse transcription quantitative PCR (RT-qPCR) approaches that use divergent or BSJ-spanning primers to selectively amplify circular molecules. In BSJ-directed RT-qPCR assays, isoform discrimination is established primarily by primer placement across the back-splice junction and the associated splice architecture. Hydrolysis probes (TaqMan probes), which contain a 5′ reporter fluorophore and a 3′ quencher, generate fluorescence when probe cleavage by the 5′-3′ exonuclease activity of Taq polymerase separates the reporter from the quencher during primer extension. This adds a second sequence-recognition event within the amplicon, improving assay stringency by reducing nonspecific signal and technical background; however, it does not independently confer isoform-level discrimination. A limitation of hydrolysis probe designs is that extremely short circRNAs (<70 nt) may not provide sufficient sequence space for efficient primer/probe placement, potentially restricting assay adaptability for shorter circular transcripts.

circRNAs are widely expressed across eukaryotic systems and have been implicated in development, cancer, oxidative stress responses, cardiovascular disease, and neurological and autoimmune disorders [3,4,5,6,7,8]. Mechanistically, circRNAs have been reported to regulate gene expression through several routes, including sequestration of microRNAs, modulation of RNA-binding protein activity, regulation of transcription, and, in some cases, translation from internal ribosome entry site-containing templates [1,9,10,11,12,13,14]. Beyond cellular transcripts, circRNAs derived from viral genomes have also been experimentally identified, and many additional candidates have been predicted by deep sequencing [15]. Viral circRNAs from Epstein–Barr virus (EBV) and Kaposi’s sarcoma-associated herpesvirus (KSHV) can promote metastasis by antagonizing tumor-suppressive cellular miRNAs [16,17], and a human papillomavirus (HPV)-derived circRNA serves as a major template for translation of the viral E7 oncoprotein [18]. We previously showed that Human Immunodeficiency Virus type 1 (HIV-1) also generates circRNAs that can sequester cellular miRNAs and enhance viral replication [19]. Together, these findings highlight both the biological relevance of viral circRNAs and the need for detection methods with sufficient isoform resolution to support mechanistic studies.

HIV-1 presents an especially complex system for circRNA analysis because its primary transcript undergoes extensive alternative splicing, generating a highly complex population of viral RNAs with substantial sequence redundancy. We previously demonstrated that HIV-1 produces 15 circRNAs through backsplicing of its primary transcript [19], in addition to more than 40 linear viral RNA species that share common exons and splice junctions. This extensive overlap limits the utility of conventional approaches such as Northern blotting and hybridization-based assays, including RNAscope-like strategies, for the discrimination of individual HIV circRNA isoforms. In this setting, BSJ-centered RT-qPCR offers a more practical route to isoform-resolved detection. Building on our prior divergent primer strategy, we developed a TaqMan probe-based qPCR platform for HIV circRNA analysis in which individual assays are designed around specific BSJs and paired with internal hydrolysis probes to increase sequence selectivity. 

The workflow also incorporates assays for total HIV RNA and a cellular housekeeping transcript to enable normalization for viral transcript abundance and RNA input quality. This strategy provides a reliable and robust approach for the sensitive and specific detection of HIV circRNAs within a highly overlapping viral transcriptome. Orthogonal circRNA validation approaches, including RNase R enrichment, LNA-based Northern blotting, direct sequencing of back-splice junction amplicons, and droplet digital PCR [20,21,22,23,24], remain important complementary tools for confirming circRNA identity and abundance. Our divergent TaqMan platform is intended as a scalable assay framework that integrates with these validation strategies, rather than replacing them.

## 2. Materials and Methods

### 2.1. Plasmids, Cell Lines, Transfections and Virus Preparations

The infectious HIV-1 clone pNL4-3 was obtained from the NIH HIV Reagent Program, Division of AIDS, NIAID, NIH: Human Immunodeficiency Virus 1 (HIV-1), Strain NL4-3 Infectious Molecular Clone (pNL4-3), ARP-114, contributed by Dr. Malcolm Martin. The RevCEM-D4 cells were obtained from the NIH HIV Reagent Program, Division of AIDS, NIAID, NIH: RevCEM-D4 Cells, ARP-13437, contributed by Dr. Alex Sigal. The HEK293 cells were obtained from the ATCC (CRL-1573), HEK293. Cells were grown and maintained following the guidelines provided by the respective repositories. HEK293 cells were transfected utilizing 50 ng of pNL4-3 plasmid DNA and Lipofectamine 2000 (Thermo-Fisher Scientifics, Waltham, MA, USA) in 24-well plates according to the manufacturer’s protocol. HEK293 cells were seeded 24 h before transfection at 50% confluence in 500 μL of D-MEM supplemented with 10% fetal calf serum and gentamicin. HEK293 cells were washed 24 h post-transfection to remove residual plasmid, and virus-enriched supernatant was harvested 72 h post-transfection. RevCEM-D4 cells were seeded 24 h before infection at 50% confluence in 24-well plates and infected using the viral preparations obtained from the HEK293 cells at multiplicity of infection (MOI) = 10. Total RNA was isolated from the infected RevCEM-D4 cells 72 h after infection.

### 2.2. circRNA Isolation and Quantification

Total RNA was extracted using a TRIzol (Thermo-Fisher Scientific) centric protocol we previously optimized for extraction of viral RNA [25]. RNA was DNase-treated with Turbo DNase (Thermo-Fischer Scientific) at 37 °C for 60 min for effective plasmid removal. RNA was reverse-transcribed using a random pd(N)6 primer and Superscript II RT (Thermo-Fischer Scientific) according to the manufacturer’s protocol. qPCR analysis of the circular and linear viral transcripts was carried out utilizing the primers shown in Supplementary Table S1. TaqMan probes were synthesized by IDT Inc. (Coralville, IA, USA). The HIV-1 circRNA specific TaqMan probes TqP-Ex2, TqP-Ex3, TqP-Tat and TqP-Rev/Nef carry the 5′ fluorophore 6-FAM (520) and the quenchers ZEN/Iowa Black FQ. The probe TqP-RPL13A carries the 5′ fluorophore HEX (555) and the quenchers ZEN/Iowa Black FQ. The probe TqP-All-HIV, used for the quantification of total viral RNA, carries the 5′ fluorophore Cy5 (668) and the quenchers TAO/Iowa Black RQ. Total HIV-1 RNA (or the housekeeping gene *RPL13A*) and a target HIV circRNA were amplified using a multiplex TaqMan qPCR assay. Conversely, for the SYBR Green qPCR assays, these targets were amplified in separate reactions. qPCRs were performed utilizing the Agilent AriaMx real-time PCR system and data analyzed using the AriaMx v2.1.1 software. For qPCRs using Taqman probes, the Brilliant II qRT-PCR kit (Agilent) was utilized, and primers and probes were diluted to a final concentration of 400 nM and 200 nM, respectively. For all other qPCR, the Green-2-Go SYBR green qPCR Kit (BioBasic) was utilized, and primers were diluted to a final concentration of 200 nM. The reaction conditions for all qPCR assays were 95 °C for 10 min, followed by 40 cycles of 95 °C for 30 s and 62 °C for 60 s. The assays were carried out in technical duplicates and data are represented as means ± SEM. RNase R digestion was performed by treating 10 μg of RNA with 0.3 μL of RNase R (NEB) at 37 °C for 15 min. A mock-digestion containing RNA and RNase R reaction buffer, but no enzyme, was incubated at the same time to serve as a negative control. The efficiency of linear RNA removal was assessed by parallel qPCR for the linear host transcript RPL13A and for the HIV-1 linear mRNAs (All-HIV). Relative circRNA abundance in infected cells and donor samples was determined using the ∆Ct method, normalized to total HIV RNA transcripts detected by the ‘All-HIV’ primer set: ∆Ct_(circRNA) = Ct_(circRNA) − Ct_(All-HIV). Where indicated, HIV levels were further normalized to the host housekeeping gene RPL13A to account for total RNA input: ∆Ct_(HIV) = Ct_(All-HIV) − Ct_(RPL13A). No RT controls are shown in the Supplementary Source Data.

### 2.3. Primary T Cells Isolation, Activation, and Infection

De-identified blood apheresis samples from healthy individuals were obtained through OneBlood (www.oneblood.org) and classified as non-human research by the Florida Atlantic University Health Sciences IRB. PBMC isolation was achieved using Ficoll-Paque Plus (MilliporeSigma) and SepMate-50 (STEMCELL) tubes following the manufacturers’ protocols. Naïve CD4+ T cells were isolated from the PBMCs by negative selection using the EasySep Human CD4+ T Cell Isolation Kit (STEMCELL Technologies) according to the manufacturer’s protocol. Prior to infection, T cells were activated for 120 h by incubating 2.5 × 10^6^ cells in 1 mL of RPMI 1640 medium supplemented with 10% heat-inactivated fetal bovine serum in 24-well plates coated with anti-CD3 antibody (BioXCell). Anti-CD28 antibody (BD bioscience) at a final concentration of 6 μg/mL, and 20 units/mL of interleukin-2 (PeproTech), were also added to each well during activation. Infections of the activated T cells were carried out in 1 mL of RPMI 1640 supplemented with 10% heat-inactivated FBS, 20 units/mL of interleukin-2, 6 μg/mL of polybrene, and an HIV-1 preparation with a MOI = 1. MOI was calculated by dividing the viral titer by the number of cells. Viral preparations were obtained by transfecting HEK293 cells with the molecular clone pNL4-3 as described above, washing the cells twice with PBS after 24 h to remove residual plasmid, and harvesting the supernatant 72 h post-transfection. The viral titer was determined using the TZM-bl reporter cell line, as previously described [26]. Some T cells were mock-infected with supernatant from mock-transfected HEK293 cells to serve as a control. T cells were washed twice with PBS 24 h post-infection to remove any residual virions and then incubated for 5 days before total RNA extraction.

## 3. Results

### 3.1. Design of a Divergent TaqMan RT-qPCR Strategy for HIV-1 circRNA Detection

**Figure 1:**
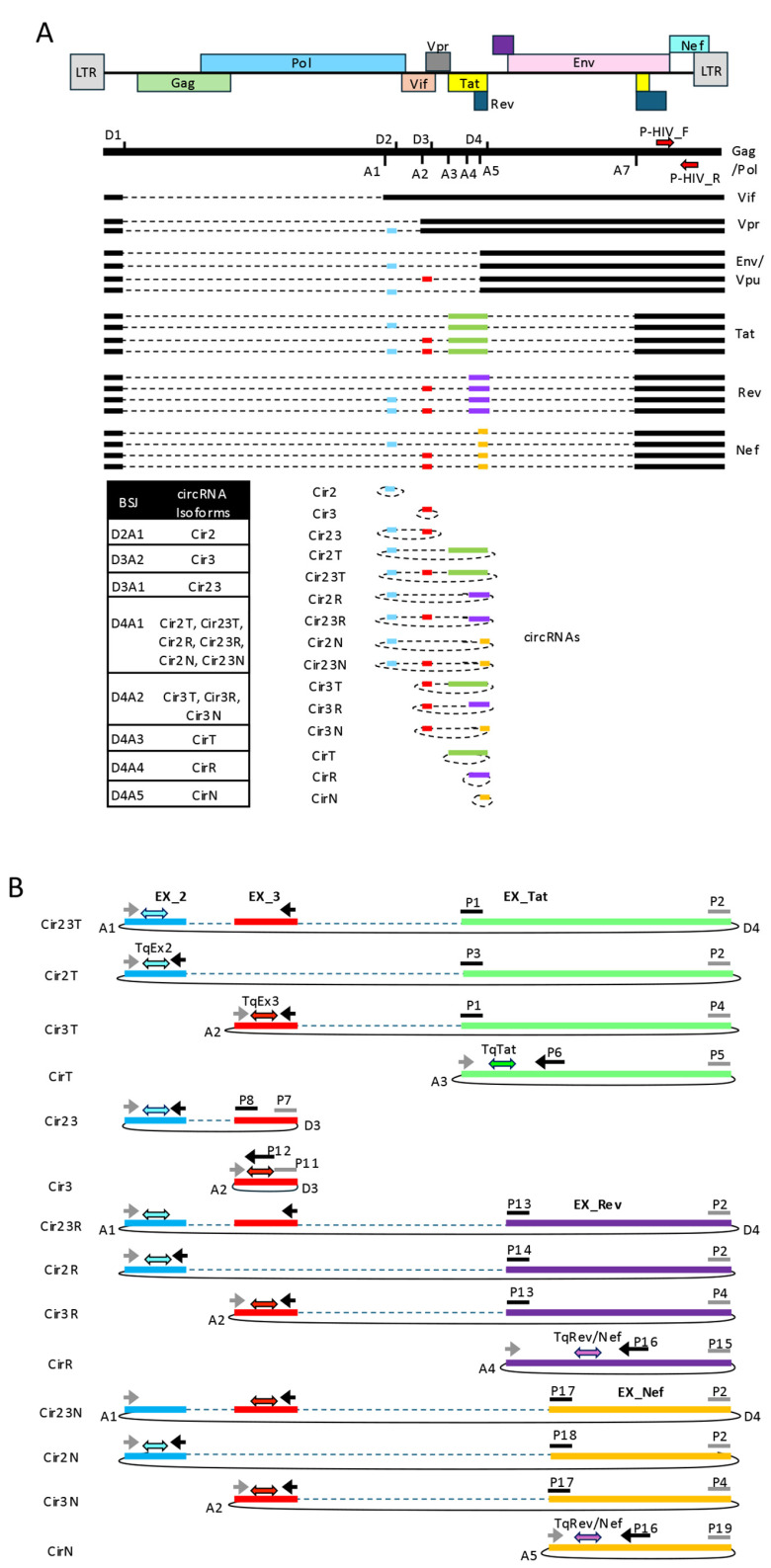
Divergent TaqMan RT-qPCR strategy for isoform-specific detection of HIV-1 circRNAs. (**A**) Schematic representation of the major alternatively spliced HIV-1 mRNAs generated through usage of the principal donor and acceptor splice sites. The 15 HIV-1 circRNA isoforms produced by backsplicing of the major donor and acceptor splice sites are also indicated. The primer set used to detect the total pool of linear HIV transcripts (P-HIV_F; P-HIV_R) is shown. (**B**) Design of the divergent TaqMan RT-qPCR assays used for HIV-1 circRNA detection. Divergent primer pairs (labeled P) were designed to amplify individual circRNA isoforms by targeting a BSJ in combination with either a canonical splice junction or a contiguous stretch of viral transcript sequence, as indicated. Primer orientation is denoted by arrows marking the 3′ end of each primer; in junction-spanning primers, the 3′ end overlaps one side of the splice junction (BSJ or linear splice junction), whereas the straight line indicates the 5′ portion of the primer extending across the opposite side of the junction. The positions of the TaqMan hydrolysis probes (labeled Tq) are indicated by double-headed arrows.

The HIV-1 transcriptome is exceptionally complex, as the combined use of major and minor donor and acceptor splice sites generates numerous linear and circular RNA isoforms that share extensive sequence overlap and often similar sizes (Figure 1A). This complexity makes the identification of individual HIV circRNAs by conventional approaches, such as Northern blotting or hybridization-based assays, challenging. In our previous work, we addressed this limitation using an isoform-specific qPCR strategy in which 15 primer sets were designed with one or both primers spanning a back-splice junction and the companion primer positioned on a canonical splice junction or contiguous stretch of transcript, enabling selective amplification of individual HIV circRNA species. Using that approach, all 15 predicted HIV circRNA isoforms were detected in infected Rev-CEM-D4 cells. Building on this framework, we developed a divergent TaqMan RT-qPCR platform in which back-splice-junction-directed amplification is coupled to exon-specific hydrolysis probes, thereby improving signal specificity and reducing nonspecific background while preserving BSJ-defined isoform discrimination. circRNA-directed probes were labeled with the 5′ fluorophore 6-FAM (520) and carried dual 3′ quenchers ZEN/Iowa Black FQ to improve signal-to-background performance. We utilized this approach to design assays for 14 of the 15 predicted HIV-1 circRNAs. The only exception was Cir2, whose very short length (50 nt) precluded the design of an efficient TaqMan probe-based amplification strategy. The TaqMan platform was practically adaptable to 14 isoforms and enabled a more stringent and scalable approach for selective HIV circRNA quantification (Figure 1B).

### 3.2. Analytical Validation of the HIV circRNA TaqMan Assay Panel

**Figure 2:**
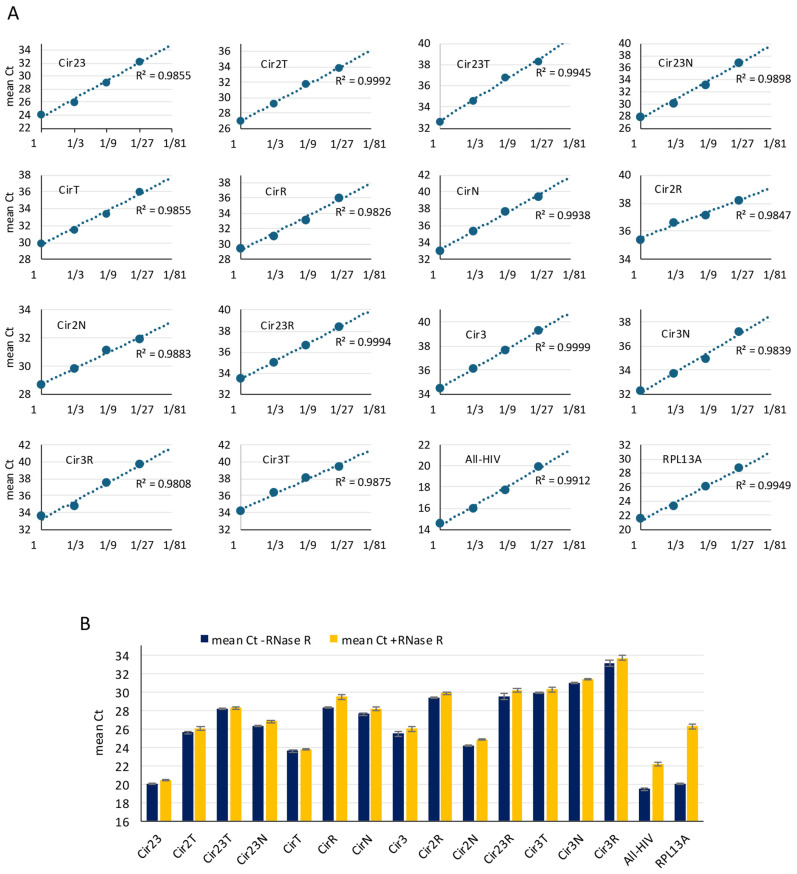
Validation of the HIV-1 circRNA TaqMan RT-qPCR assay panel. (**A**) Standard curves for each HIV-1 circRNA assay generated by amplifying 3-fold serial dilutions of cDNA prepared from total RNA isolated from Rev-CEM-D4 cells infected with the HIV-1 NL4-3 strain. Control assays detecting total linear HIV transcripts (All-HIV) and the endogenous reference linear RNA transcript RPL13A were analyzed in parallel. The coefficients of determination (R^2^) are indicated. (**B**) RNase R validation of the HIV-1 circRNA assays. cDNA synthesized from untreated RNA or RNase R-treated RNA was analyzed by RT-qPCR to assess resistance of the circular targets to exonuclease digestion. HIV circRNAs remained readily detectable after RNase R treatment, whereas the linear controls All-HIV and RPL13A showed marked Ct increases, consistent with selective degradation of linear RNAs. Data are represented as means ± SEM. No RT controls (NRT) are shown in the Supplementary Source Data Table.

We next evaluated the analytical performance of the primer-probe sets using cDNA generated from total RNA isolated from HIV-1 NL4-3-infected Rev-CEM-D4 cells. Standard curves generated from 3-fold serial dilutions showed a strong linear relationship between input cDNA and Ct value across the complete assay panel (Figure 1A). All assays produced reliable Ct values across at least a 27-fold dilution range, and the coefficients of determination (R^2^) ranged from 0.980 to 0.999 across the validated assays, indicating robust linear amplification over the tested dilution series. These data support the suitability of the TaqMan assay for quantitative detection across a wide dynamic range and show that the panel performs reproducibly despite the sequence complexity of the HIV splicing landscape (Figure 2A).

Two additional TaqMan control assays were incorporated into the workflow to support quantitative comparisons across samples. An All-HIV assay, designed to detect the total pool of linear HIV transcripts, used a Cy5 (668)-labeled probe with TAO/Iowa Black RQ quenchers and was used to normalize individual circRNA signals to total HIV mRNA abundance. In parallel, a housekeeping assay for RPL13A used a HEX (555)-labeled probe with the ZEN/Iowa Black FQ quenchers to monitor host transcript recovery and to normalize viral transcript measurements to RNA input and extraction quality. Together, this multiplex-compatible design enabled circRNA-specific quantification while controlling both for variation in viral transcriptional output and for sample-to-sample differences in RNA quality.

To further test whether the detected amplicons behaved as expected for circular templates, we compared the Ct values obtained by amplifying the cDNA obtained from the RNA samples before and after RNase R treatment, a 3′ exoribonuclease which digests linear RNAs but not circular RNAs. All circRNAs were detected with a minimal (<0.5) increase in Ct following RNase R digestion, consistent with persistence of circular RNA templates after exonuclease treatment, whereas linear RNAs such as All-HIV and RPL13A, included as sensitivity controls for RNase R susceptibility, showed a remarkable increase in Ct (3–6 cycles) (Figure 2B). The RNase R assay supports the reliability of the TaqMan assay for the detection of HIV circRNAs. 

### 3.3. TaqMan Assays Show Higher Specificity than SYBR Green Assays

**Figure 3:**
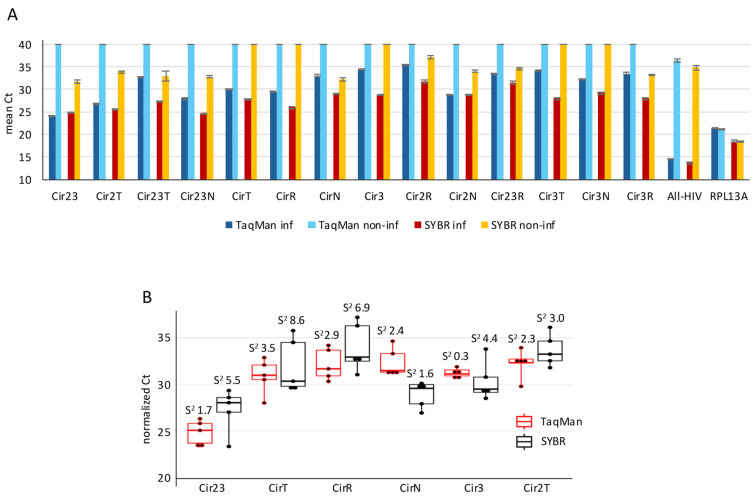
Comparison of divergent TaqMan and SYBR Green qPCR assays for HIV-1 circRNA detection. (**A**) cDNA from HIV-1-infected and non-infected Rev-CEM-D4 cells was analyzed by qPCR using the indicated circRNA-specific TaqMan primer-probe sets or the corresponding SYBR Green primer pairs. Amplification profiles were compared to assess assay specificity and relative background signal. Ct values were normalized for the total HIV RNA abundance in the infected samples and relative to the endogenous RPL13A transcript abundance in the non-infected samples. Total data are represented as means ± SEM. (**B**) Performance of TaqMan and SYBR Green assays in donor-derived samples. CD4+ T cells were purified from five healthy donors, activated with anti-CD3/CD28 antibodies and IL2 and infected with HIV-1 (NL4-3 clone). cDNA synthesized from total RNA extracted from the infected CD4+ T cells was amplified using circRNA-specific TaqMan primer-probe sets or the corresponding SYBR Green primer pairs. Box plots show Ct values normalized for the total HIV RNA abundance for each donor (individual dots). The variance (s^2^) for each circRNA target is indicated for both types of assay. No RT controls (NRT) are shown in the Supplementary Source Data Table.

We then compared the performance of the TaqMan assays with corresponding SYBR Green qPCR assays in infected and non-infected Rev-CEM-D4 cells. In infected cells, both chemistries detected HIV-derived targets; however, the TaqMan assays showed markedly improved specificity in non-infected controls. For most circRNA targets, non-infected samples analyzed by TaqMan remained at or near the maximal Ct value, whereas the matched SYBR Green reactions produced appreciable amplification for nine of the 14 circRNAs in non-infected samples, indicating higher background signal with the dye-based approach. This pattern was especially notable because the RPL13A control amplified in both infected and non-infected samples, confirming comparable cDNA input and demonstrating that the reduced background was specific to the HIV-targeted TaqMan design, rather than a global loss of amplifiability. Together, these data indicate that coupling divergent primers to exon-specific hydrolysis probes substantially improves discrimination between true HIV circRNA signal and nonspecific amplification (Figure 3A).

We also compared assay consistency in infected, donor-derived, CD4+ T cell samples by examining normalized Ct distributions for representative HIV circRNAs. The TaqMan assays showed lower sample variance than the corresponding SYBR Green assays (TaqMan average s^2^ = 2.2 vs. SYBR average s^2^ = 5). CirN was the main exception, for which SYBR Green showed slightly lower variance than TaqMan (1.6 vs. 2.4). Thus, across most targets tested in this primary cell comparison, the TaqMan format produced tighter distributions and more reproducible normalized measurements than SYBR Green. These findings, together with the infected/non-infected comparison, support the conclusion that the TaqMan platform offers a lower background fluorescence and improved measurement consistency under the conditions tested (Figure 3B).

## 4. Discussion

The present study establishes a divergent TaqMan RT-qPCR platform for isoform-resolved detection of HIV circRNAs in the context of the technical problem posed by the HIV-1 transcriptome: an unusually dense splicing architecture in which many circular and linear RNAs share extensive sequence identity. The assay panel combines amplification across the back-splice junctions and linear splice junctions with an internal hydrolysis probe, so the divergent TaqMan design provides an additional sequence-verification step requiring probe hybridization within the correctly amplified product, thereby reducing fluorescence generated from nonspecific amplification products. Consistent with this design, the assays showed strong linearity across serial dilutions, retained signal after RNase R treatment, and outperformed matched SYBR Green assays primarily by reducing background amplification in non-infected controls and by improving measurement consistency across most targets tested. Together, these data indicate that probe-based detection provides a practical advantage for HIV circRNA analysis in a transcriptome dominated by highly overlapping linear and circular RNA species [19,21].

The utility of this assay design likely extends beyond the NL4-3 molecular clone used in this study. Analyses of subtype B transmitted/founder HIV-1 isolates have demonstrated that major splice donor and acceptor sites, along with overall viral splicing patterns, are highly conserved across independent isolates [27]. Since HIV-1 circRNA biogenesis relies on backsplicing between these canonical sites, this conserved splicing architecture suggests that the combinations required for circular RNA production are also preserved across diverse strains. Consequently, HIV circRNA production may represent a fundamental feature of HIV-1 biology, implying that the divergent TaqMan assay framework described here could be adapted for detection across genetically distinct isolates, requiring only minimal sequence-level optimization of primers and probes.

These findings are significant because accurate circRNA quantification depends on selective interrogation of the back-splice junction, particularly in systems in which circular and linear transcripts share nearly all internal sequences. Current best-practice guidelines emphasize that circRNA measurements should incorporate multiple independent validation steps and should not rely on a single analytical feature alone [21]. In this regard, the RNase R results presented here provide important biochemical support for the assay design, while the improved specificity over SYBR Green further supports the utility of hydrolysis probe chemistry for resolving closely related viral RNA isoforms [21,28]. The inclusion of the All-HIV and RPL13A control assays also strengthens the workflow by enabling normalization to total viral transcription and RNA input quality, respectively, thereby improving the interpretability of circRNA measurements across infected-cell and donor-derived samples.

One limitation of the current panel is that Circ2 could not be adapted to the TaqMan format because its 50 nt length does not provide sufficient sequence space for efficient probe-based assay design. This constraint is not unique to HIV circRNAs but rather reflects a broader limitation of hydrolysis probe strategies when the circular target is extremely short. Future optimization of this target may require alternative probe chemistries or junction-focused detection formats. More generally, additional validation, including direct sequencing of amplified back-splice junctions, may further strengthen circRNA assignment in settings where very low-abundance species are being investigated [20,21].

A particularly important future direction will be adaptation of this assay set to reverse transcription droplet digital PCR (RT-ddPCR). Because ddPCR enables partition-based absolute quantification without reliance on standard curves, it is well suited for low-copy targets and limited-input samples, conditions that are likely to be relevant for HIV circRNA analysis in primary CD4+ T cells, latency models, and clinical specimens [22]. The existing TaqMan assay architecture should facilitate this transition, as probe-based chemistries are directly compatible with ddPCR workflows. Implementation of RT-ddPCR could facilitate absolute quantification following formal analytical validation, including the determination of LoD, LoQ, precision, and partition cluster separation. This precise quantification of individual HIV circRNAs would enable a direct correlation between circRNA abundance and total viral RNA output on a per-sample basis. Such an approach would likely improve inter-assay comparability and expand the utility of HIV circRNA measurements in mechanistic and translational studies. Future ddPCR development should, however, include formal analytical validation in accordance with digital minimum information for publication of quantitative real-time PCR experiment (MIQE) principles, including determination of the limit of detection, limit of quantification, cluster separation, and assay precision across low-copy inputs [22,29].

## Data Availability

The original contributions presented in this study are included in the article/Supplementary Material. For further inquiries, please contact the corresponding author.

## Supplementary Materials

The following supporting information can be downloaded at: https://www.mdpi.com/article/10.3390/mps9030077/s1, Table S1: Primers and probes utilized in the study. Supplementary Source Data: Source data for the qPCR performed.
