# Supplementary material for: A Divergent TaqMan RT-qPCR Strategy for Isoform-Resolved Detection of HIV-1 Circular RNAs
Source: Methods Protoc. 2026 May 13;9(3):77. doi: 10.3390/mps9030077 (PMC13214776; doi:10.3390/mps9030077)
Supplement: Supplementary file 1 [file mps-09-00077-s001.zip › Supplemental Table S1_REV.pdf]

Supplemental Table S1

Primers/Probes List

Target	Forward primer	Reverse Primer	Probe
HIV Cir3	cHIV-P11 GCAGGACATAACAAGAATCTGC	cHIV-P12 TTGATATTCACACCTAGGAC	TqP-Ex3-FMFQ AGAAATACCATATTAGGACGTATAG
HIV Cir23	cHIV-P7 GACATAACAAGGGACAGCAG	cHIV-P8 TGCTTGATATTCACACCTAG	TqP-Ex2-FMFQ AGATCCAGTTTGGAAAGGACCAGCA
HIV CirT	cHIV-P5 TCTATCAAAGCAAATTGGGT	cHIV-P6 ATGCTTCCAGGGCTCTAGTC	TqP-ExTat-FMFQ CTCGACAGAGGAGAGCAAGAAATG
HIV Cir2T	cHIV-P2 GCTTCTCTATCAAAGCAGGAC	cHIV-P3 GTCGACACCCAATTCTTTCCA	TqP-Ex2-FMFQ AGATCCAGTTTGGAAAGGACCAGCA
HIV Cir23T	cHIV-P2 GCTTCTCTATCAAAGCAGGAC	cHIV-P1 CGACACCCAATTCTTGTTATG	TqP-Ex2-FMFQ AGATCCAGTTTGGAAAGGACCAGCA
HIV Cir2R	cHIV-P2 GCTTCTCTATCAAAGCAGGAC	cHIV-P14 CATAGGAGATGCCTAAGGCTT	TqP-Ex2-FMFQ AGATCCAGTTTGGAAAGGACCAGCA
HIV Cir23R	cHIV-P2 GCTTCTCTATCAAAGCAGGAC	cHIV-P13 ATAGGAGATGCCTAAGGCTTG	TqP-Ex2-FMFQ AGATCCAGTTTGGAAAGGACCAGCA
HIV Cir2N	cHIV-P2 GCTTCTCTATCAAAGCAGGAC	cHIV-P18 CTCCGCTTCTTCCTTTCCAG	TqP-Ex2-FMFQ AGATCCAGTTTGGAAAGGACCAGCA
HIV Cir23N	cHIV-P2 GCTTCTCTATCAAAGCAGGAC	cHIV-P17 CTGTCTCCGCTTCTTCCTTGT	TqP-Ex2-FMFQ AGATCCAGTTTGGAAAGGACCAGCA
HIV Cir3T	cHIV-P4b TCTATCAAAGCAGGACAGCAG	cHIV-P1 CGACACCCAATTCTTGTTATG	TqP-Ex3-FMFQ AGAAATACCATATTAGGACGTATAG
HIV Cir3R	cHIV-P4b TCTATCAAAGCAGGACAGCAG	cHIV-P13 ATAGGAGATGCCTAAGGCTTG	TqP-Ex3-FMFQ AGAAATACCATATTAGGACGTATAG
HIV Cir3N	cHIV-P4c CTCTATCAAAGCAAATCTGC	cHIV-P17 CTGTCTCCGCTTCTTCCTTGT	TqP-Ex3-FMFQ AGAAATACCATATTAGGACGTATAG
HIV CirR	cHIV-P15 TCTCTATCAAAGCACCTTAG	cHIV-P16 TTGATGAGTCTGACTGTTCTG	TqP-ExRev/Nef-FMFQ AAGCGGAGACAGCGACGAAGAGCT
HIV CirN	cHIV-P19 TCTCTATCAAAGCAGAAGAAG	cHIV-P16 TTGATGAGTCTGACTGTTCTG	TqP-ExRev/Nef-FMFQ AAGCGGAGACAGCGACGAAGAGCT
All-HIV	P-HIV-Fw GCTATTCGCCACATACCTAG	P-HIV-Rv AGGCCATCCAATCACACTAC	TqP-RPL13A-HXFQ CTATAAGATGGGTGGCAAGTGGTC
RPL13A	RPL13A-Fw AGCCAGAAGACTGATTGGAG	RPL13A-Rv AGTGCTTGCACATTCTAACAG	TqP-AllHIV-CY5FQ CATCTGTTGGACTTTCCACCTGGT
